# Serum biomarkers VEGF-C and IL-6 are associated with severe human Peripheral Artery Stenosis

**DOI:** 10.1186/s12950-015-0095-y

**Published:** 2015-08-18

**Authors:** Jiexia Chen, Lei Han, Xiaoyan Xu, Haiqin Tang, Hongyan Wang, Bin Wei

**Affiliations:** The First Affiliated Hospital of Anhui Medical University, Ji Xi Road, Hefei, Anhui 230022 China; Institute of Biochemistry and Cell Biology, Shanghai Institutes for Biological Sciences, Chinese Academy of Sciences, 320 Yue Yang Road, Shanghai, 200031 China; Cancer Research Center, Xuhui Central Hospital, Shanghai Clinical Center, Chinese Academy of Sciences, Shanghai, 200031 China; State Key Laboratory of Virology, Wuhan Institute of Virology, Chinese Academy of Sciences, Xiao Hong Shan Road, Wuhan, Hubei 430071 China

**Keywords:** Inflammation, VEGF-C, IL-6, Monocytes, and Peripheral artery disease

## Abstract

**Background:**

Emerging reports propose possible biomarkers that are related to inflammation, nutrition and lipid parameters for detection of the progression of atherosclerotic plaques, peripheral artery disease (PAD) and particularly peripheral artery stenosis (PAS). However, it remains unclear which biomarkers in serum are associated with the severity of PAS.

**Findings:**

In this study, we measured serum levels of inflammatory biomarkers along with lipid and nutritional parameters in 53 patients who suffered different degrees of PAS. Serum concentrations of vascular endothelial growth factor-c (VEGF-C) and IL-6 (Interleukin 6) were significantly increased in patients showing moderate or severe PAS. Furthermore, the number of blood monocytes from PAS patients was significantly increased, which showed elevated adhesion to plate-coated fibrinogen. Compared to healthy subjects, freshly isolated or LPS (lipopolysaccharide)-stimulated blood monocytes from PAS patients could produce VEGF-C and IL-6 at higher levels.

**Conclusions:**

Our study suggests that the increased number of blood monocytes might play key roles during the development of severe PAS, which enhance adhesion at the local narrowed peripheral artery and secret high levels of VEGF-C and IL-6. We suggest that serum concentrations of VEGF-C and IL-6 might be used as biomarkers for diagnosis severe PAS in combination with clinical imaging examination.

**Electronic supplementary material:**

The online version of this article (doi:10.1186/s12950-015-0095-y) contains supplementary material, which is available to authorized users.

## Findings

### Introduction

In patients with atherosclerotic peripheral artery stenosis (PAS), the degree of stenosis and the structure of plaque are important risk factors for cardiovascular events and adverse limb events. Current optimal morphological evaluation of PAS aims to make an accurate diagnosis through invasive and non-invasive imaging techniques [1]. However, simple methods, such as blood test of biomarkers, with potential usage for high-throughput analysis and diagnosis of the severity of PAS are not fully developed. Previous studies support that inflammation and innate immune cells including monocytes or macrophages play important roles during the development and progression of PAS [2, 3]. Various risk factors are used as potential criteria for detection of subclinical PAS [2, 4–6], such as inflammatory biomarkers including C-reactive protein (CRP), fibrinogen, lipoprotein, cholesterol, along with blood glucose and triglyceride.

Recently, we reported that in response to bacterial infection, macrophages increased the expression of soluble vascular endothelial growth factor C (VEGF-C) and the surface receptor VEGFR-3 (vascular endothelial growth factor receptor-3, also termed Flt-4) in a Toll-like receptor-4 (TLR4)-dependent manner. Further, serum VEGF-C expression was substantially increased in sepsis patients, suggesting serum VEGF-C as a new biomarker for diagnosis of sepsis [7]. Previous findings have demonstrated that upon binding to VEGF-C, the tyrosine kinase receptor VEGFR-3 is activated [7–9]. Interestingly, others also reported that VEGF-C is chemotactic for recruitment of macrophages or T lymphocytes into target organs under pathological conditions [10, 11]. A link has been established between pathogenesis of atherosclerosis and the VEGF family members, such as VEGF-A (also termed VEGF) [12]. In addition, concentrations of VEGF-A are enhanced and its binding to VEGFR-1 have been linked with sepsis in humans and mice [13, 14]. Although we provided evidence that serum VEGF-C levels are associated with human or murine sepsis, it remains unknown whether serum VEGF-C is linked to PAS severity. In this study, we examined the number of immune cells and a series of inflammatory risk factors in blood from moderate and severe PAS patients. We found that serum VEGF-C and IL-6 concentrations were significantly increased in severe PAS patients compared with healthy subjects. Moreover, the number of blood monocytes in PAS patients was increased with elevated adhesion to plate-coated fibrinogen. Blood monocytes in PAS patients also produced higher levels of VEGF-C and IL-6 (Interleukin 6). It suggests that accumulation of adherent monocytes at local narrowed peripheral artery might promote production of inflammatory risk factors to increase the severity of PAS.

## Material and methods

### Enroll of patients and healthy subjects

Multi-slice computed tomography (CT) was used to analyze the grade of stenosis and morphology of soft or hard plaque. According to the grade of stenosis and plaque morphology, patients were divided into two groups: Thirty-one patients with atherosclerotic peripheral artery stenosis >50 % but <80 %, twenty-two patients with stenosis ≥80 %. Twenty-seven healthy subjects were enrolled. The exclusion criteria include a recent infection, a recent acute coronary syndrome, heart failure (NYHA class III of IV), stenosis with a cause other than atherosclerosis, renal failure, and liver disease. The levels of plasma triglycerides, HDL and LDL cholesterol, fasting blood glucose, fibrinogen, and white blood cell counts were determined by routine laboratory methods.

### Preparation and immunostaining of PBMCs

Peripheral blood mononuclear cells (PBMCs) from healthy people and PAS patients were isolated by density-gradient centrifugation with Ficoll 1.077 (0850494, MP). PBMCs were immunostained with anti-CD11b and anti-F4/80 antibodies (11–0113 and 14–4801, eBioscience) in PBS with 1 % BSA. Samples were analysed on a BD Accuri C6 flow cytometry.

### Monocytes isolation and LPS stimulation

Human monocytes were isolated from peripheral blood using a modified Recalde method [15]. Briefly, 50 μL of 1.54 M NaCl was added into 10 mL PBS to suspend 3×10^7^ PBMCs. After incubation at 37 °C for 10 min, an additional 100 μL of 1.54 M NaCl was added, followed by a third addition of 1.54 M NaCl (100 μL) 10 min later. Next, the cells were diluted by the addition of 15 mL of hypertonic PBS [1.54 M NaCl were added to PBS in a ratio of 1:36 (v/v)] and mixed by pipeting up and down. 10-mL aliquots were dispensed into 50-mL centrifuge tubes, and mixed with an additional 15 mL of hypertonic PBS. Each tube was then underlayered with 15 mL of hypertonic Ficoll. Hypertonic Ficoll was prepared by adding 2.8 mg of crystalline NaCl to each mL of Ficoll. The tubes were centrifuged at 600 g for 15 min at 25 °C. The monocytes were found at and below the interface in a broad band, which was removed (approximately 15 ml). The contents of each tube were diluted to 40 mL with PBS and centrifuged at 600 g for 15 min. The cell pellets were washed twice in 5 mM EDTA in PBS to remove platelets and resuspended in DMEM. Then, monocytes were immunostained with anti-CD14 antibody (557154, BD) and the average purity was 70 % by FACS analysis. Monocytes were seeded into 96-well tissue culture plates at a density of 1.0×l0^5^ per well and stimulated with or without LPS (100 or 300 ng/mL) for 24 h in DMEM.

### Adhesion assay

A 24-well tissue culture plate was coated with fibrinogen (F3879, Sigma) (10 μg/mL, dissolved in PBS) and incubated at 4 °C overnight. PBMCs were seeded into the plate at a density of 5×10^5^ per well and cultured for 16 h in DMEM without serum. After washed three times with PBS, cells were stained with anti-CD11b antibody to mark monocytes. Images were captured with Olympus BX51 microscope and adherent monocytes were counted.

### Detection of cytokine concentrations

The concentrations of interleukin 6 (IL-6) and vascular endothelial growth factor C (VEGF-C) in plasma and supernatants were determined using ELISA kits (BMS213INST & BMS297/2, eBioscience) according to the manufacturer’s instructions.

### RNA isolation and quantitative real-time reverse transcriptase-polymerase chain reaction

Total RNA was isolated from PBMCs using TRIzol Reagent (DP405, TIANGEN), then converted to cDNA using M-MLV reverse transcriptase (2641A, TAKARA). Quantitative reverse transcriptase-polymerase chain reaction (qRT-PCR) was performed on a CFX-96 machine (Bio-rad) using SYBR Green master mix (DBI Bioscience). Values were normalized for *β-actin* mRNA levels. The average mRNA levels of IL-6, IL-1β and VEGF-C in PBMCs from healthy subjects were set as 1.00 and those from PAS patients were calculated as the fold change.

### Statistical analysis

Data are presented as mean ± SD. A two-tailed Student’s *t*-test was used to compare two groups. ANOVA and Dunnett’s test were used to analyze difference among three groups. For all test, a P value of 0.05 or less was considered statistically significant.

## Results

### PAS patients enhance the number of blood monocytes and serum concentrations of IL-6 or VEGF-C

**Fig. 1:**
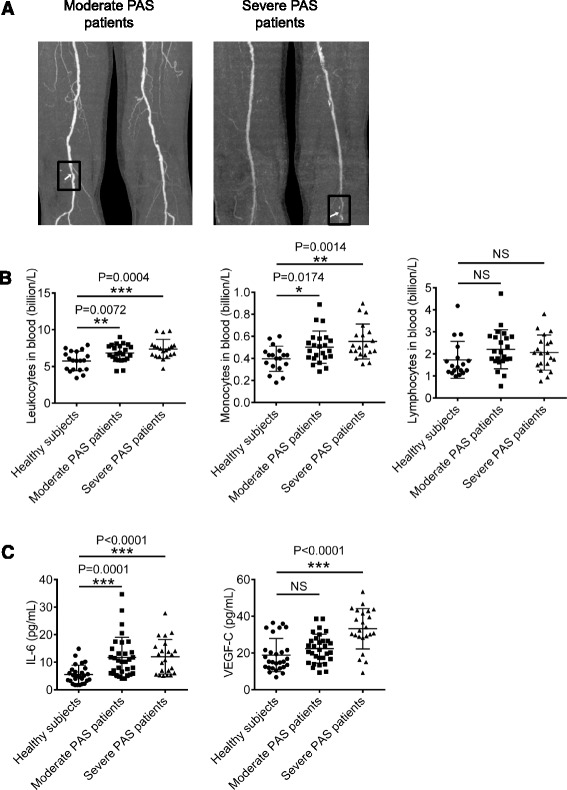
PAS patients enhance the number of blood monocytes and serum concentrations of IL-6 and VEGF-C. **a** The grades of artery stenosis and morphology of soft or hard plaque of PAS patients were analyzed with CT. **b** Blood was collected from 18 healthy subjects, 23 moderate PAS patients and 20 severe PAS patients. The number of leukocytes, monocytes and lymphocytes were calculated by Blood Cell Counter. **c** Protein levels of serum IL-6 and VEGF-C from 27 healthy subjects, 31 moderate PAS patients and 22 severe PAS patients were examined with ELISA

Multi-slice computed tomography (CT) was used to analyze the grades of stenosis and morphology of soft or hard plaque of patients. According to the grades of stenosis and morphology of plaque, patients were classified into two groups and compared with age-matched healthy subjects. Patients with atherosclerotic PAS 50 % ~ 80 % were defined as moderate PAS patients, and patients with PAS ≥80 % were defined as severe PAS (Fig. 1a, the boxed area represents a typical narrowed artery). Compared with healthy subjects, the number of leukocytes in blood was significantly increased in moderate or severe PAS patients (Fig. 1b, left panel, healthy vs. moderate PAS, P = 0.0072; healthy vs. severe PAS, P = 0.0004). Particularly, the number of monocytes in blood from moderate or severe PAS patients was higher than that of healthy subjects, (Fig. 1b, middle panel, healthy vs. moderate PAS, P = 0.0174; healthy vs. severe PAS, P = 0.0014). In contrast, both moderate and severe PAS patients had the comparable number of lymphocytes in blood compared with healthy subjects (Fig. 1b, right panel).

We next examined which risk factors might show association with the grades of PAS. Serum concentrations of IL-6, VEGF-C, cholesterol, high density lipoprotein (HDL), low density lipoprotein (LDL), very low density lipoprotein (VLDL), blood glucose and triglyceride from PAS patients and healthy subjects were tested. Interestingly, IL-6 and VEGF-C concentrations were substantially increased in severe PAS patients compared with those of healthy subjects (Fig. 1c, left panel, healthy vs. severe PAS, P < 0.0001; right panel, healthy vs. severe PAS, P < 0.0001). The levels of IL-6 were also significantly increased in moderate PAS patients compared with those of healthy subjects (Fig. 1c, left panel, healthy vs. moderate PAS, P < 0.0001). Of note, moderate PAS patients displayed similar levels of blood glucose, triglyceride, HDL, LDL, VLDL, and cholesterol compared with those of healthy subjects (Additional file 1: Figure S1A, B, C and D). Moreover, severe PAS patients had normal levels of blood glucose, triglyceride, HDL and VLDL compared with those of healthy subjects (Additional file 1: Figure S1A, B, C and D). These data indicate that the elevated number of monocytes, serum levels of IL-6 and VEGF-C are associated with severe PAS.

### Blood monocytes in PAS patients produce higher levels of IL-6 and VEGF-C

**Fig. 2:**
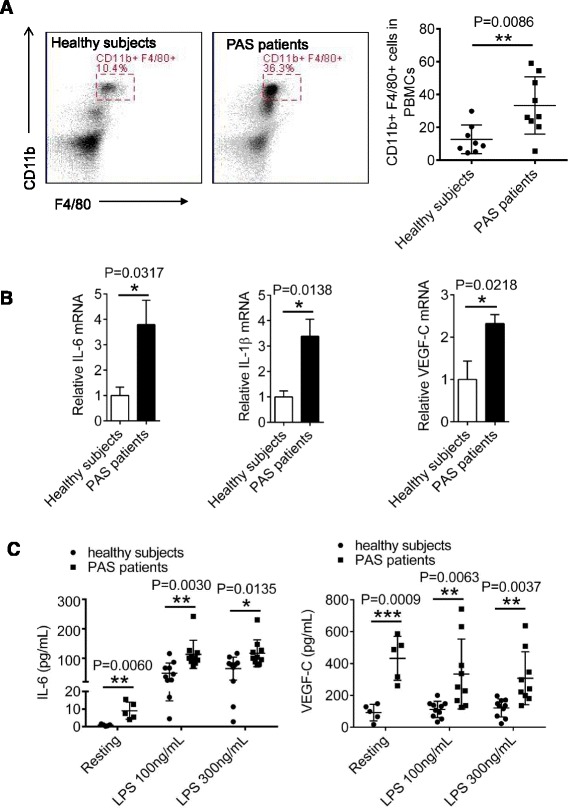
Blood monocytes in PAS patients produce inflammatory cytokines and VEGF-C. **a** PBMCs were isolated from blood of 8 healthy subjects and 9 PAS patients by density-gradient centrifugation with Ficoll (1.077), followed by staining with FITC-conjugated anti-CD11b and APC-conjugated anti-F4/80 for flow cytometer analysis. **b** Relative mRNA levels IL-6, VEGF-C or IL-1β in PBMCs from healthy subjects and PAS patients were tested with qRT-PCR. **c** Freshly isolated monocytes from healthy subjects and PAS patients were either untreated (n = 6) or stimulated with different doses of LPS for 24 h (n = 10), followed by detection of IL-6 and VEGF-C concentrations from the supernatants by ELISA. Data presented are representative of three replicated experiments

To evaluate the role of monocytes in secretion of inflammatory cytokines, PBMCs in peripheral blood were prepared from 8 healthy subjects and 9 PAS patients and the percentage of monocytes was measured using flow cytometry. Consistent with our previous observation (Fig. 1b), the percentage of monocytes was increased in PAS patients (Fig. 2a, P = 0.0086). Furthermore, the mRNA levels of IL-6, IL-1β and VEGF-C from PBMCs were increased in PAS patients than those of healthy subjects (Fig. 2b, left panel, P = 0.0317; middle panel, P = 0.0138; right panel, P = 0.0218). We next isolated blood monocytes from PAS patients and healthy subjects. Concentrations of IL-6 and VEGF-C from supernatants of fresh isolated monocytes or LPS-treated monocytes were detected by ELISA. In resting stage, blood monocytes from PAS patients enhanced concentration of IL-6 and VEGF-C compared with those of healthy subjects, despite that IL-6 was secreted at low concentrations (Fig. 2c left panel, P = 0.0060; right panel, P = 0.0009). In response to LPS (lipopolysaccharide) stimulation, blood monocytes significantly enhanced IL-6 production. Compared to healthy controls, LPS-stimulated monocytes from PAS patients produced higher concentrations of IL-6 and VEGF-C (Fig. 2c, left panel, LPS 100 ng/mL, P = 0.0030, LPS 300 ng/mL, P = 0.0135; right panel, LPS 100 ng/mL, P = 0.0063, LPS 300 ng/mL, P = 0.0037). Together, our data suggest that the increased number of monocytes in PAS patients produce higher levels of VEGF-C and IL-6 or IL-1β.

### Blood monocytes in PAS patients enhance adhesion

**Fig. 3:**
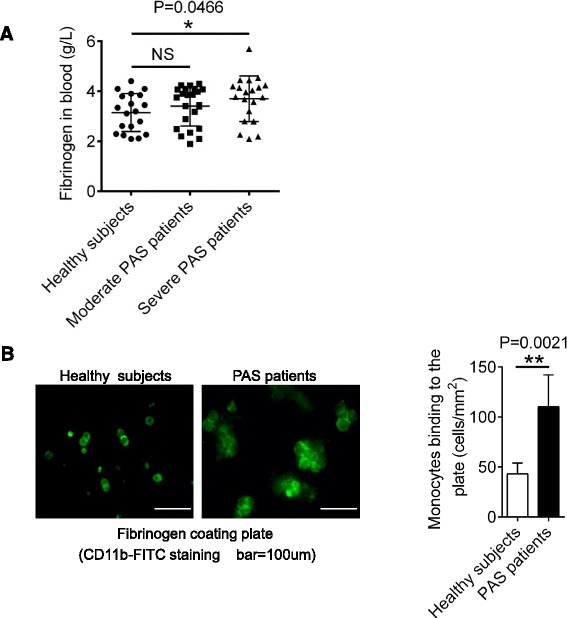
Blood monocytes in PAS patients enhance adhesion. **a** The levels of fibrinogen in blood from 18 healthy subjects, 23 moderate PAS patients and 20 severe PAS patients were tested. **b** PBMCs from 3 healthy subjects and 3 PAS patients were cultured on the plate coated with fibrinogen for 16 h. Adherent monocytes were marked with anti-CD11b-FITC and counted with Olympus BX51 microscope. Data presented are representative of three replicated experiments

Interestingly, similar to the result of VEGF-C, we observed that the protein levels of fibrinogen in severe PAS patients were increased compared to healthy subjects (Fig. 3a, P = 0.0466). We next prepared PBMCs from PAS patients and healthy subjects, and assessed their adhesion ability to fibrinogen. After adherent to the fibrinogen-coated plate, monocytes were stained with FITC-conjugated anti-CD11b. Monocytes from PAS patients displayed spreading or enlarged morphology in response to fibrinogen stimulation (Fig. 3b, left panel). Moreover, increased number of monocytes from PAS patients were adherent to fibrinogen compared with those from healthy subjects (Fig. 3b, right panel, P = 0.0021). These data suggest that increased number of monocytes in PAS patients adhere more strongly to fibrinogen-containing surface.

## Discussion

Emerging observations show that monocytes play important roles in the initiation, propagation, and progression of PAS [16]. This study provides direct evidence that peripheral monocytes from PAS patients produced higher levels of inflammatory cytokines including IL-6 and VEGF-C, which were associated with severe PAS. Moreover, serum concentrations of fibrinogen were also significantly elevated in severe PAS patients. Numerous studies indicate soluble fibrinogen in the blood stream promotes clot formation [17]; excess fibrinogen exaggerates inflammation and destroys the endothelium, which promotes atherosclerosis [18]. Therefore, persistently elevated fibrinogen levels are a major risk factor for atherosclerosis, which predict heart attacks and strokes with exceptional accuracy. In addition, the inflammatory factors, such as IL-6, TNF-α, significantly contribute to the development, progression and destabilization of atherosclerotic plaques [19]; production of fibrinogen might be enhanced by inflammatory process associated with atherosclerosis [20]. However, few reports suggest the participation of fibrinogen and IL-6 in the development of PAS. Our study proposes that serum fibrinogen, IL-6 and VEGF-C concentrations might be used to diagnosis severe PAS. Compared with imaging techniques, such as Doppler Ultrasonography, Magnetic Resonance Angiography (MRA) or Computed Tomography Angiography (CTA), blood or serum biomarkers are easy to be detected by simple methods. Therefore, detecting serum fibrinogen, IL-6, VEGF-C enables less cost for high-throughput analysis of severe PAS in high-risk populations.

Apart from the role in inflammatory process, atherosclerosis plaques have been shown to contain fibrinogen degradation products [21], which may act as chemoattractants for leukocytes. Furthermore, VEGF-C has also been reported to mediate cell migration [10]. We observed elevated number of monocytes, but not lymphocytes, in peripheral blood stream, which was correlated to the severity of PAS. Notably, peripheral monocytes from PAS patients increased adhesion to fibrinogen-coated surface, whereas fibrinogen binds to integrin α5β1. The receptors for VEGF-C are VEGFR-3 and VEGFR-2, which express on monocytes, macrophages [7, 11] as well as on lymphatic or vascular endothelial cells [22, 23]. Previous studies have shown that exogenous VEGF-C treatment enhances transmigration ability of macrophages *in vitro* [11] and VEGF-C/VEGFR-3 signaling cooperates with PI3K to guide T lymphocyte migration *in vivo* [10]. It might be expected that increased VEGF-C production in severe PAS patients might trigger VEGFR-2/3 signaling in monocytes to enhance integrin-mediated adhesion to vascular wall for subsequent accumulation. We and others previously identified the intracellular signaling proteins ADAP (Adhesion- and Degranulation-promoting Adapter Protein, also known as FYB or SLAP-130), SKAP-HOM (SKAP55 homologue, also known as SKAP-2 or SKAP55R) for enhancing integrin-mediated immune cell adhesion [24–32]. Since VEGF-C/VEGFR-3 signaling activates the PI3K/AKT pathway in macrophages [7], and the ADAP/SKAP-HOM complex expresses in macrophages that is also linked to PI3K [33], it is interesting to further investigate whether VEGF-C/VEGFR-3 signaling cooperates with the ADAP/SKAP-HOM complex to regulate integrin activation and monocytes/macrophage adhesion. Together, these data suggest that blood monocytes might stick and accumulate at narrowed local artery in response to high concentrations of serum fibrinogen and VEGF-C in PAS, then become one main source to produce inflammatory cytokines and exacerbate the severity of PAS.

## Additional file

Additional file 1: Figure S1. Serum concentrations of glucose (A), triglyceride (B), HDL, LDL, VLDL (C) and cholesterol (D) from 18 healthy subjects, 23 moderate PAS patients and 20 severe PAS patients were examined. (PPT 732 kb)
