# Supplementary figures and images for: Serum biomarkers VEGF-C and IL-6 are associated with severe human Peripheral Artery Stenosis
Source: J Inflamm (Lond). 2015 Aug 18;12:50. doi: 10.1186/s12950-015-0095-y (PMC4538759; doi:10.1186/s12950-015-0095-y)

## Slide 1
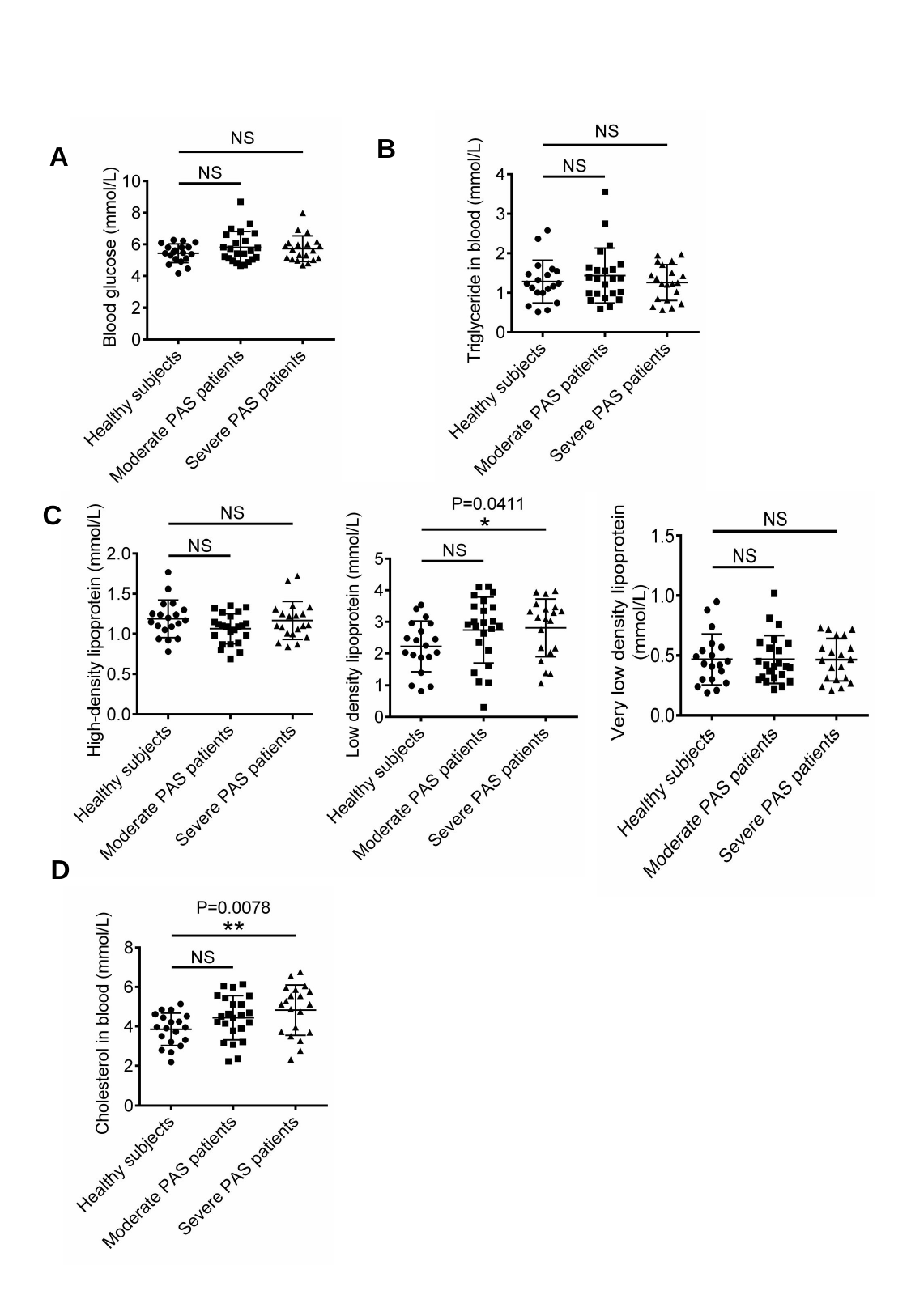

B
A
C
D

Supplement: Additional file 1: Figure S1. — Serum concentrations of glucose (A), triglyceride (B), HDL, LDL, VLDL (C) and cholesterol (D) from 18 healthy subjects, 23 moderate PAS patients and 20 severe PAS patients were examined. (PPT 732 kb) [file 12950_2015_95_MOESM1_ESM.ppt]
